# BRCA1/ATF1-Mediated Transactivation is Involved in Resistance to PARP Inhibitors and Cisplatin

**DOI:** 10.1158/2767-9764.CRC-21-0064

**Published:** 2021-11-12

**Authors:** Shino Endo, Yuki Yoshino, Matsuyuki Shirota, Gou Watanabe, Natsuko Chiba

**Affiliations:** 1 Department of Cancer Biology, Institute of Aging, Development, and Cancer, Tohoku University, Sendai, Japan.; 2 Department of Cancer Biology, Tohoku University Graduate School of Medicine, Sendai, Japan.; 3 Laboratory of Cancer Biology, Graduate School of Life Sciences, Tohoku University, Sendai, Japan.; 4 Division of Interdisciplinary Medical Science, Tohoku University Graduate School of Medicine, Sendai, Japan.; 5 Tohoku Medical and Pharmaceutical University, Sendai, Japan.

## Abstract

Homologous recombination (HR)-deficient cells are sensitive to PARP inhibitors through a synthetic lethal effect. We previously developed an HR activity assay named Assay of Site-Specific HR Activity (ASHRA). Here, we evaluated the HR activity of 30 missense variants of BRCA1 by ASHRA and found that several BRCA1 variants showed intermediate HR activity, which was not clearly discerned by our previous analyses using a conventional method. HR activity measured by ASHRA was significantly correlated with sensitivity to olaparib. However, cells expressing the severely HR-deficient BRCA1-C61G variant were resistant to olaparib, and resistance was dependent on high expression of activating transcription factor 1 (ATF1), which binds to BRCA1 and activates the transcription of target genes to regulate cell proliferation. The BRCA1-C61G variant bound to ATF1 and stimulated ATF1-mediated transactivation similar to wild-type BRCA1. High expression of ATF1 conferred resistance to olaparib and cisplatin activating BRCA1/ATF1-mediated transcription without affecting HR activity in BRCA2-knockdown or RAD51-knockdown cells, but not in BRCA1-knockdown cells. These results suggest that ASHRA is a useful method to evaluate HR activity in cells and to predict the sensitivity to PARP inhibitors. The expression level of ATF1 might be an important biomarker of the effect of PARP inhibitors and platinum agents on HR-deficient tumors with the BRCA1-C61G variant or alteration of non-BRCA1 HR factors such as BRCA2 and RAD51.

**Significance:**

ASHRA could evaluate HR activity in cells and predict the sensitivity to PARP inhibitors. High expression level of ATF1 may predict the resistance of BRCAness tumors with alterations of non-BRCA1 HR factors to PARP inhibitors and platinum agents.

## Introduction

Small-molecule inhibitors of PARP have been developed for the treatment of various cancers, including breast, ovarian, pancreatic, and prostate cancers (1). PARP functions in various DNA damage repair pathways and in DNA replication (2). PARP inhibitors impair the repair of DNA single-stranded breaks (SSB), which results in the generation of more cytotoxic DNA double-stranded breaks (DSB), and trap PARP at SSBs, thereby preventing repair (3). Because PARP contributes to the restart of stalled replication forks, PARP inhibition promotes the collapse of replication forks (4, 5). Homologous recombination (HR) contributes to the repair of DSBs, PARP trapping, and collapsed forks (3). Therefore, PARP inhibitors induce synthetic lethality in cells with HR deficiency (HRD).

Genetic alterations of HR factors including *BRCA1/2* cause hereditary cancer such as hereditary breast and ovarian cancer syndrome (6). In addition, HRD is observed in various sporadic cancers (7). The phenotype of tumors that carry germline *BRCA1/2* mutations is called BRCAness, which is found in some sporadic tumors with methylation of *BRCA1/2* or inactivation of HR factors other than BRCA1/2, resulting in HRD (8). Approximately 50% of ovarian cancers exhibit HRD. Alterations of *BRCA1/2* are found in more than 50% of HR-deficient ovarian cancers, whereas alterations of *non-BRCA1/2* HR factors, including *RAD51C* or *PALB2*, are present in the remaining cases (9).

Genetic analysis of *BRCA1/2* genes and evaluation of HRD by genomic scarring assays are used clinically to stratify patients who would benefit from treatment with a PARP inhibitor. However, these stratification strategies have certain limitations. First, a number of variants of uncertain significance of *BRCA1/2* have been reported. Second, various resistance mechanisms induce primary and acquired resistance to PARP inhibitors (10), and the genomic scarring assay does not detect resistance to PARP inhibitors mediated by certain mechanisms, including revertant mutations of HR factors, which restore HR activity (11–13). In addition, recent clinical studies report that PARP inhibitors are beneficial for patients regardless of *BRCA1/2* status and HRD according to the genomic scaring assay (14–16).

**FIGURE 1:**
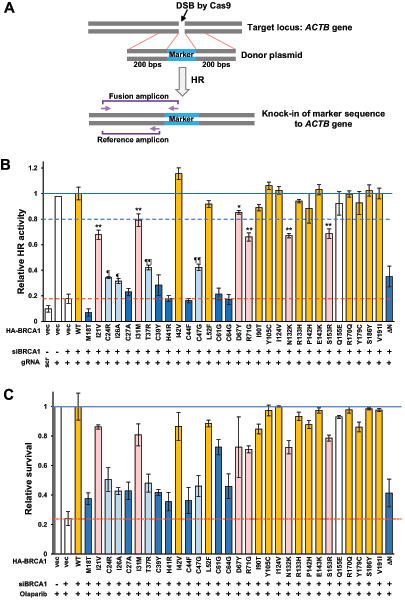
Evaluation of the HR activity and sensitivity to olaparib of 30 BRCA1 missense variants. **A,** Schematic of ASHRA and the design of primers used in this study. **B,** HR activities of HeLa cells expressing BRCA1 missense variants were measured by ASHRA. The amount of the marker sequence was quantified by the ΔΔ*C*_t_ method using the positive control samples (transfected with gRNA against the *ACTB* gene and control siRNA) as a reference. Values were normalized to the samples rescued by BRCA1-WT as 1. Data are the mean of at least three independent experiments. Error bars show SEM. Variants presented in yellow or pale pink bars and blue or pale blue bars were categorized by the DR-GFP assay as HR proficient and HR deficient, respectively (20). The blue solid line and blue broken line indicate levels of 1.0 and 0.8, respectively. The red broken line indicates the level of samples with BRCA1 knockdown. scr: scramble gRNA was used as the negative control for ASHRA. * and **: versus WT, *P* < 0.05 and *P* < 0.01, respectively. ¶ and ¶¶: versus BRCA1-knockdown samples, *P* < 0.05 and *P* < 0.01, respectively. **C,** Relative survival of HeLa cells expressing BRCA1 missense variants. Cells were treated with 0.5 μmol/L olaparib for 5 days after transfection of siRNA and HA-BRCA1 expression vectors. Values were normalized to control sample (transfected with control siRNA) as 1. Data are expressed as the mean ± SEM of three independent experiments. The bar colors of variants are the same as in A. The blue solid line and red broken line indicate 1.0 and 0.24 (value of BRCA1-knockdown samples), respectively.

We recently developed an assay called Assay for Site-specific HR Activity (ASHRA) to evaluate cellular HR activity (Fig. 1A; ref. 17). In ASHRA, a DSB is specifically created in the endogenous target locus of the genome by the Cas9 endonuclease. When the DSB is repaired by HR using the donor vector containing a marker sequence flanked by the two arms homologous to the target locus as a template, the marker sequence is knocked-in to the target locus. Consequently, HR activity can be evaluated by quantifying the knock-in frequency by quantitative PCR.

Several methods have been used to evaluate the HR activity of cells. The DR-GFP assay uses cells in which a pair of GFP expression cassettes is integrated into the genome. Thus, the DR-GFP assay does not target endogenous gene loci. In addition, because the DR-GFP assay detects the GFP protein expressed after repair by HR, it does not directly detect HR products at the DNA level. Several methods were developed to evaluate the HR activity of cells using the CRISPR/Cas9 system (18, 19). Certo and colleagues used Cas9 to cut the GFP-mCherry cassette integrated into the genome (18). This method enables simultaneous evaluation of HR and non-homologous end joining activities, whereas it also targets the exogenous sequence. Pinder and colleagues knocked-in the GFP sequence into the *LMNA* gene, and evaluated HR activity by analyzing LMNA-GFP–positive cells (19). Although this method targets endogenous loci, it cannot target un-transcribed genes or detect HR activity at the DNA level.

In contrast, ASHRA targets the endogenous gene locus and directly detects the HR products at the DNA level by quantitative PCR. Because ASHRA does not require the expression of target genes, it could target any genomic locus of interest. Furthermore, ASHRA is performed by transient transfection of two vectors for the expression of gRNA and Cas9 and for the HR donor sequence. Thus, ASHRA does not require the establishment of a stable cell line.

In several reports, results obtained using DR-GFP assay seem to be qualitative rather than quantitative under certain experimental conditions (20–23). We previously showed that the HR activity of BRCA1 variants, the I26A missense variant and the amino (N)-terminal deletant, measured by ASHRA showed a better correlation with the sensitivity of cells expressing those variants to PARP inhibitors than that measured by the DR-GFP assay (17). This led us to speculate that measurement of HR activity by ASHRA provides an accurate prediction of the sensitivity to PARP inhibitors.

In this article, we used ASHRA to evaluate the HR activity of BRCA1 missense variants, and analyzed the correlation between the HR activity and the sensitivity of cells expressing the variants to a PARP inhibitor, olaparib. ASHRA detected the partial HRD of some BRCA1 variants. The HR activity of BRCA1 variants measured by ASHRA was significantly correlated with the sensitivity of cells expressing the variants to olaparib. In addition, we found that high expression of activating transcription factor 1 (ATF1), which interacts with BRCA1, might contribute to resistance to olaparib and cisplatin in HR-deficient cells.

## Materials and Methods

### Cell Lines and Culture

HeLa, MCF7, HEK-293T, MCF10A, and BT-549 cells were purchased from ATCC. HeLa, MCF7, and HEK-293T cells were maintained in DMEM (Nissui Pharmaceutical) supplemented with 8% FBS (Biowest) in an atmosphere with 5% CO_2_. MCF10A cells were maintained in DMEM/F-12 (Sigma-Aldrich) supplemented with 5% FBS, 20 ng/mL human EGF (PeproTech), 10 μg/mL human insulin, and 0.5 μg/mL hydrocortisone (Tokyo Chemical Industry) in an atmosphere with 5% CO_2_. BT-549 cells were maintained in RPMI1640 (Nissui pharmaceutical) supplemented with 8% FBS and 10 μg/mL human insulin (Sigma-Aldrich) in an atmosphere with 5% CO_2_. Cell line identities were verified using the GenomeLab Human STR Primer set (Beckman Coulter). Cells were routinely stained by Hoechst 33342 and no mycoplasma contamination was suspected. Cells were used for experiments within 20 passages from obtaining.

### Plasmid and siRNA

Plasmid vectors for ASHRA were described previously (17). Expression vectors for HA-BRCA1-WT or variants were described previously (20, 21). To construct an expression vector for FLAG-ATF1, the coding sequence of ATF1 was amplified by PCR using the primers 5′-CAAGTCGACATGGAAGATTCCCACAA-3′ and 5′-ACTGCGGCCGCCAACACTTTTATTGGA-3′ and cloned into the XhoI/NotI site of the pCY4B-FLAG vector (24, 25). The sequence of siRNA against the 3′-untranslated region (UTR) of BRCA1 was 5′-GCUCCUCUCACUCUUCAGU-3′ (20). Dicer-substrate siRNAs against the 3′-UTR of ATF1 (hs.Ri.ATF1.13.1), BRCA2 (hs.Ri.BRCA2.13.3) and RAD51 (hs.Ri.RAD51.13.1) were purchased from Integrated DNA Technologies.

### Transfection

For the cotransfection of siRNA and plasmid, the Trans-IT X2 dynamic delivery system (Mirus Bio) was used according to the manufacturer's instructions. For the transfection of ASHRA vectors, polyethyleneimine MAX (Polysciences) was used as described previously (17).

### Western Blotting

Western blotting was performed according to the conventional method with minor modifications (17). Following antibodies were used; a polyclonal anti-BRCA1 antibody specific for residues 1528–1863 of BRCA1, anti-BRCA2 (sc-8326, Santa Cruz Biotechnology), anti-RAD51 (GTX100469, GeneTax), anti-ATF1 (HPA055069, Sigma-Aldrich), anti-α-tubulin (DM1A, Merck), anti-β-actin (6D1, Wako Purechemicals), anti-MBP (E8032, New England Biolabs).

### HR Activity Assay

The measurement of HR activity by ASHRA was described previously (17). In brief, cells grown in 3.5 cm dishes were transfected with siRNA against BRCA1 and the HA-tagged BRCA1 expression vector. On the next day, the donor vector (Addgene ID: #169798) and the expression vector for gRNA and Cas9 (Addgene ID: #169795 and #169796; 0.5 μg each) were cotransfected into the cells. After 48 hours of incubation, genomic DNA was extracted using the Blood Genomic DNA Extraction Mini Kit (Favorgen). Quantitative PCR was performed on a CFX96 Touch Real-time PCR detection system (Bio-Rad) using GoTaq qPCR master mix (Promega). Quantification of the knocked-in allele and control allele by qPCR was performed using the following primer sets: 5′-GTCCTGCTGGAGTTCGTGACCG-3′ and 5′-GTGCAATCAAAGTCCTCGGC-3′ for the knocked-in allele, and 5′-AGTTGCGTTACACCCTTTCTTG-3′ and 5′-GTGCAATCAAAGTCCTCGGC-3′ for the control allele. The relative quantity of the knocked-in allele was calculated by the ΔΔ*C*_t_ method.

### Drug Sensitivity Assay

Cells were seeded in 96-well plates and transfected with siRNA and the HA-BRCA1 expression vectors. After 2 hours of transfection, 0 or 0.5 μmol/L olaparib (Adooq Bioscience) was added. For HeLa or MCF10A cells, cell viability was measured using the PrestoBlue assay (Thermo Fisher Scientific) according to the manufacturer's instructions after 5 days of incubation. For MCF7 cells, the medium was changed to fresh medium with olaparib on day 3, and cell viability was measured on day 5. For BT-549 cells, cells were treated with 4.0 μmol/L olaparib for 5 days with medium change on day 3.

### Quantification of mRNA Level by RT-PCR

The sequences of primers used to quantify ATF1-target genes were described by Tian and colleagues (26). Total mRNA was extracted from cells by ISOGEN II (Nippon Gene) and reverse-transcribed using the PrimeScript II first-strand cDNA Synthesis Kit (Takara Bio). Quantitative PCR was performed on a CFX96 Touch Real-time PCR detection system using GoTaq qPCR master mix. The relative expression of genes of interest was calculated using the ΔΔ*C*_t_ method with GAPDH as an internal reference.

### Statistical Analysis

Statistical analysis was performed using JMP 14 software (SAS Institute Inc). Graphs were constructed using Excel 2016 (Microsoft). Statistical comparisons between two different samples were made using a two-tailed Welch *t* test. A *P* value of <0.05 was considered significant.

### Data Availability

The data generated in this study are available within the article and its Supplementary Data files.

## Results

### ASHRA Detects Partial HRD of BRCA1 Missense Variants

To validate the accuracy of ASHRA for detecting sensitivity to PARP inhibitors, 30 BRCA1 missense variants in the N-terminal region including the RING domain that we previously analyzed by the DR-GFP assay (20, 21), were reevaluated using ASHRA. HeLa cells were cotransfected with siRNA against the 3′-UTR of BRCA1 mRNA and HA-tagged BRCA1 (HA-BRCA1) expression vectors. The expression level of each variant was confirmed by Western blotting (Supplementary Fig. S1A). As shown in Fig. 1B, the results of ASHRA showed that BRCA1 variants possessed various levels of HR activity: yellow or pale pink bars indicate HR-proficient variants, whereas blue or pale blue bars indicate HR-deficient variants according to previous DR-GFP assays (20, 21). Six BRCA1 variants (I21V, I31M, D67Y, R71G, N132K, and S153R shown by pale pink bars in Fig. 1B) showed significantly lower HR activity than wild-type BRCA1, although these variants were categorized as HR-proficient by the DR-GFP assay (20, 21). Four variants (C24R, I26A, T37R, and C47G, shown by pale blue bars in Fig. 1B) showed significantly higher HR activity than BRCA1-knockdown cells despite the classification of these variants as HR deficient by the DR-GFP assay (20).

To investigate the significance of the intermediate HR activity determined by ASHRA, we analyzed the correlation between the HR activity of BRCA1 variants and the survival rate of cells expressing the variants after exposure to olaparib. Olaparib was used at 0.5 μmol/L according to the dose-survival curve (Supplementary Fig. S1B). The HR activity of the variants measured by ASHRA seemed to correlate with the survival rates of HeLa cells expressing the BRCA1 variants (Fig. 1C). These data suggest that ASHRA could detect moderate changes of HR activity.

### HR Activity Measured by ASHRA Linearly Correlates with the Sensitivity to Olaparib

**FIGURE 2:**
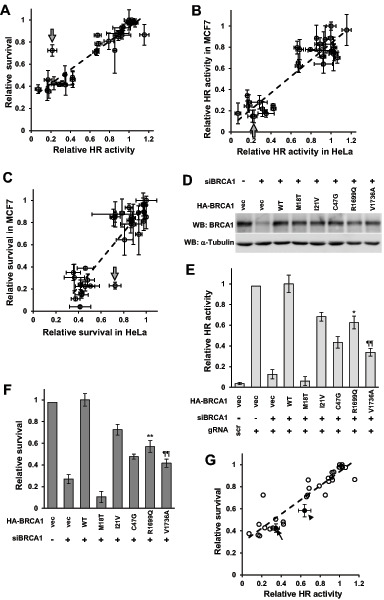
Correlation between HR activity and sensitivity to olaparib. **A,** Relative HR activities by ASHRA and relative survival under 0.5 μmol/L olaparib in HeLa cells were plotted. Error bars show SEM. Correlation coefficient (*R*^2^) = 0.88. The broken line indicates a regression line. The arrow indicates BRCA1-C61G. To test for outliers, studentized residuals from values predicted by the regression formula were calculated. **B,** Relative HR activities by ASHRA in HeLa and MCF7 cells were plotted. Error bars show SEM. *R*^2^ = 0.84. The broken line indicates a regression line. The arrow indicates BRCA1-C61G. **C,** Relative survival in response to 0.5 μmol/L olaparib in HeLa and MCF7 cells was plotted. Error bars show SEM. *R*^2^ = 0.81. The broken line indicates a regression line. The arrow indicates BRCA1-C61G. **D,** HeLa cells were transfected as indicated, and whole-cell lysates were analyzed by Western blotting. **E,** HeLa cells were transfected as indicated and HR activity was measured by ASHRA. Data are expressed as the mean ± SEM of three independent experiments. *: versus WT, *P* < 0.05. ¶¶: versus BRCA1-knockdown samples, *P* < 0.01. **F,** HeLa cells were transfected as indicated and treated with 0.5 μmol/L olaparib for 5 days. Data represent the mean ± SEM of three independent experiments. **: versus WT, *P* < 0.01. ¶¶: vs. BRCA1-knockdown samples, *P* < 0.01. **G,** The HR activity by ASHRA and sensitivity to olaparib of BRCA1-R1699Q and BRCA1-V1736A are plotted on the graph of **A**. The black dots represent R1699Q and V1736A. The arrowhead and arrow indicate BRCA1-R1699Q and BRCA1-V1736A, respectively. Error bars represent SEM. Error bars of variants shown in **A** are omitted.

To investigate whether HR activity determined by ASHRA was quantitatively correlated with the sensitivity of cells to PARP inhibitors, the correlation between the HR activity of BRCA1 variants and the survival of cells expressing these variants after treatment with olaparib was analyzed by linear regression. In HeLa cells, HR activity determined by ASHRA showed a significant linear correlation with the sensitivity to olaparib (Fig. 2A; *P* < 0.001, *R*^2^ = 0.88). The experiments were repeated using a breast cancer cell line, MCF7 (Supplementary Fig. S2A and S2B), which showed similar results (*P* < 0.001, *R*^2^ = 0.87). HR activity measured by ASHRA and survival rate after treatment with olaparib in HeLa cells was consistent with those in MCF7 cells (Fig. 2B and C).

To determine the performance of ASHRA as a quantitative assay, we measured the HR activity of the BRCA1-R1699Q and -V1736A pathogenic variants, which are associated with an intermediate risk of breast and ovarian cancer (27–30). The results of ASHRA indicated that BRCA1-R1699Q and -V1736A had 63% and 34% HR activity relative to wild-type BRCA1, respectively (Fig. 2D and E). Consistent with this, the survival rate of cells expressing these variants indicated partial sensitivity to olaparib (Fig. 2F and G).

### The BRCA1-C61G Variant Confers Resistance to Olaparib in Cells with High ATF1 Expression

Among 30 missense variants analyzed, only the C61G variant (indicated by arrows in Fig. 2A–C) showed apparent discordance between HR activity and sensitivity to olaparib in HeLa cells, but not in MCF7 cells. The studentized residual of the C61G variant in HeLa cells was >3 and it was considered as an outlier (Fig. 2A). BRCA1-C61G was severely HR deficient, as measured by ASHRA in both HeLa and MCF7 cells. This is consistent with previous reports in which the variant was analyzed by the DR-GFP assay (20, 22). Despite showing severe HRD, HeLa cells expressing BRCA1-C61G were partially but significantly resistant to olaparib, whereas MCF7 cells expressing BRCA1-C61G were highly sensitive to olaparib. Both C61G and C64G are variants at the zinc-binding residues of the RING domain that cause similar functional defects in the binding activity to BARD1, E3 ubiquitin ligase activity, and HR activity (Supplementary Table S1; refs. 20, 31–34). Indeed, BRCA1-C64G had minimal HR activity when measured by ASHRA, and conferred high sensitivity to olaparib in both HeLa and MCF7 cells (Figs. 1B, 1C, 2B, and 2C). These data led us to speculate that BRCA1-C61G confers resistance to olaparib in HeLa cells, independent of its HR activity.

**FIGURE 3:**
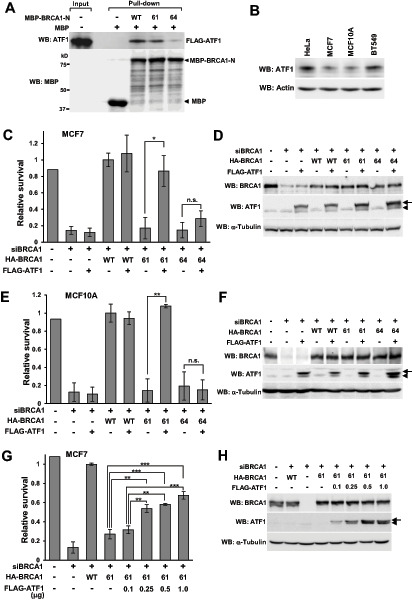
Resistance to olaparib induced by ATF1 overexpression in ATF1-low cells. **A,** HeLa cells were transfected with FLAG-ATF1 and lysed 48 hours after transfection. The lysate containing the FLAG-ATF1 protein was incubated with MBP, MBP-BRCA1-1-304-WT, BRCA1-C61G, or BRCA1-C64G and pulled down using amylose resin. Aliquots containing 1/100 of the lysate used for pulldown were loaded on the input lane. **B,** Whole-cell lysates of HeLa, MCF7, MCF10A, and BT-549 cells were analyzed by Western blotting. **C,** MCF7 cells were transfected as indicated and treated with 0.5 μmol/L olaparib for 5 days. WT, 61, and 64 indicate BRCA1-wild type, BRCA1-C61G, and BRCA1-C64G, respectively. Data represent the mean ± SEM of three independent experiments. *, *P* < 0.05; n.s., not significant. **D,** Whole-cell lysates of the samples in **C** were analyzed by Western blotting. Arrowhead: endogenous ATF1; Arrow: FLAG-ATF1. **E** and **F,** Experiments were performed as described in **C** and **D** using MCF10A cells. **, *P* < 0.01; n.s., not significant. Arrowhead: endogenous ATF1; Arrow: FLAG-ATF1. **G,** MCF7 cells were transfected as indicated and treated with 0.5 μmol/L olaparib for 5 days. WT and 61 mean BRCA1-wild type and BRCA1-C61G, respectively. Data represent the mean ± SEM of three independent experiments. **, *P* < 0.01; ***, *P* < 0.001. **H,** Whole-cell lysates of the samples in **G** were analyzed by Western blotting. Arrowhead: endogenous ATF1; Arrow: FLAG-ATF1.

Houvras and colleagues reported that BRCA1 interacts with, functions as a transcriptional coactivator of ATF1, and that the BRCA1-C61G variant retains the activity, whereas the C64G variant does not (35). ATF1 is a transcription factor belonging to the c-AMP response element-binding protein/activating transcription factor (CREB/ATF) family that activates the transcription of various genes to regulate cell proliferation and survival (26, 36). As shown in Fig. 3A, BRCA1-C61G weakly interacted with ATF1, whereas BRCA1-C64G did not. This suggests that ATF1 is involved in the different responses to olaparib between HeLa and MCF7 cells. We therefore analyzed the expression of ATF1 in these cells, and the results showed that ATF1 was expressed at considerably higher levels in HeLa cells than in MCF7 cells (Fig. 3B).

To investigate the role of ATF1 in the resistance to olaparib, we analyzed the effect of overexpression of ATF1 on the sensitivity to olaparib in MCF7 cells, which express low levels of endogenous ATF1 (Fig. 3C and D). Overexpression of FLAG-tagged ATF1 (FLAG-ATF1) did not significantly affect cell survival after treatment with olaparib in BRCA1-knockdown control cells or in cells expressing BRCA1-WT or BRCA1-C64G. In contrast, when FLAG-ATF1 was overexpressed in BRCA1-C61G–expressing cells, the MCF7 cells became markedly resistant to olaparib. Similar results were obtained using another ATF1-low cell line, MCF10A, which is derived from normal human mammary epithelia (Fig. 3B, E, and F). The effect of ATF1 on higher cell survival in BRCA1-C61G–expressing cells was correlated with the higher expression of ATF1 in MCF7 cells. Not that there is a correlation between increasing levels of ATF1 expression and increasing levels of cell survival. (Fig. 3G and H). These data suggest that high expression of the ATF1 protein confers resistance to olaparib in cells expressing BRCA1-C61G.

**FIGURE 4:**
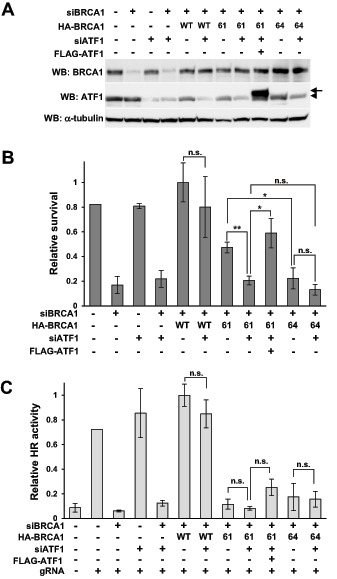
Contribution of endogenous ATF1 to olaparib resistance in ATF1-high cells. **A,** HeLa cells were transfected as indicated, and whole-cell lysates were analyzed by Western blotting. Arrowhead: endogenous ATF1; Arrow: FLAG-ATF1. **B,** HeLa cells were transfected as indicated and treated with 0.5 μmol/L olaparib for 5 days. WT, 61, and 64 indicate BRCA1-wild type, BRCA1-C61G, and BRCA1-C64G, respectively. Data are expressed as the mean ± SEM of three independent experiments. *, *P* < 0.05; **, *P* < 0.01; n.s., not significant. **C,** HeLa cells were transfected as indicated, and HR activity was measured by ASHRA. Data are expressed as the mean ± SEM of three independent experiments. n.s.: not significant.

To examine whether high expression of endogenous ATF1 contributes to the olaparib resistance of HeLa cells expressing BRCA1-C61G, ATF1 was knocked down using siRNA against the 3′-UTR of *ATF1* mRNA, and the sensitivity to olaparib was analyzed. ATF1 knockdown significantly increased the sensitivity of cells expressing BRCA1-C61G to olaparib, whereas it slightly, but not significantly, increased the sensitivity of cells expressing BRCA1-WT or BRCA1-C64G (Fig. 4A and B). The sensitivity to olaparib was similar between cells expressing BRCA1-C61G and BRCA1-C64G when ATF1 was knocked down. Exogenously expressed FLAG-ATF1 rescued the decreased survival of BRCA1-C61G–expressing cells induced by ATF1 knockdown (Fig. 4B). Knockdown of ATF1 did not affect HR activity (Fig. 4C) or cell-cycle distribution under the present experimental conditions (data not shown).

The triple-negative breast cancer cell line BT-549 is resistant to PARP inhibitors including olaparib (37). ATF1 was expressed at a high level in BT-549, as well as in HeLa cells (Fig. 3B). Consistent with our observations in HeLa cells, BT-549 cells expressing BRCA1-C61G were more resistant to olaparib than BT-549 cells expressing BRCA1-C64G (Supplementary Fig. S3A and S3B). In addition, ATF1 knockdown significantly increased the sensitivity of BT-549 cells expressing BRCA1-C61G to olaparib, whereas it slightly increased the sensitivity of cells expressing BRCA1-WT or BRCA1-C64G. Expression of FLAG-ATF1 rescued the decreased survival of BRCA1-C61G–expressing cells induced by ATF1 knockdown (Supplementary Fig. S3B). The HR activity of BRCA1-C61G and BRCA1-C64G was markedly low in BT-549 cells, consistent with the observation in HeLa and MCF7 cells (Supplementary Fig. S3C). In BT-549 cells, knockdown of endogenous ATF1 seemed to decrease HR activity and increased sensitivity to olaparib (Supplementary Fig. S3B and S3C).

### BRCA1-C61G Retains the Ability to Enhance ATF1-Regulated Transcription

**FIGURE 5:**
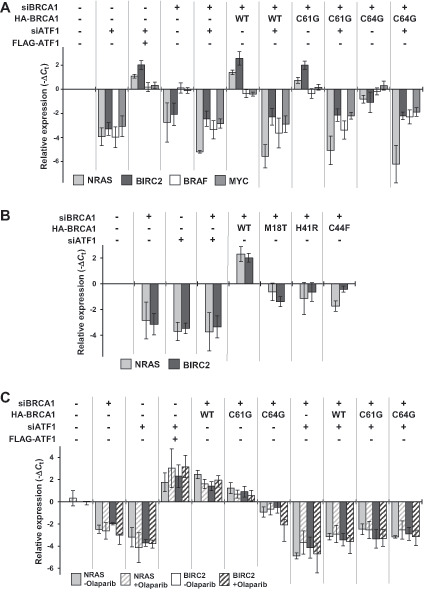
BRCA1-wild-type and BRCA1-C61G activates ATF1-regulated transcription. **A,** HeLa cells were transfected as indicated. Cells were harvested 72 hours after transfection, and total mRNA was extracted for qRT-PCR. Data are expressed as the mean ± SEM of four independent experiments. **B,** mRNA levels of *NRAS* and *BIRC2* were quantified by qRT-PCR in HeLa cells transfected as indicated. Samples were prepared as in **A**. Data represent the mean ± SEM of four independent experiments. **C,** HeLa cells were transfected as indicated and treated with or without 0.5 μmol/L olaparib for 72 hours. Experiments were repeated as well as **A**. Data represent the mean ± SEM of four independent experiments.

To investigate whether the difference in the sensitivity to olaparib between cells expressing BRCA1-C61G and BRCA1-C64G depends on the function of BRCA1 as a coactivator of ATF1-mediated transcription, we quantified the mRNA levels of *NRAS*, *BIRC2*, *MYC*, *and BRAF*, which are regulated by ATF1 (ref. 26; Fig. 5A). The mRNAs of *NRAS* and *BIRC2* were expressed only when both ATF1 and BRCA1 were expressed. BRCA1 knockdown downregulated *NRAS* and *BIRC2*, and this effect was rescued by expression of BRCA1-WT and -C61G in the presence of endogenous ATF1, but not by expression of BRCA1-C64G. The mRNA expression of *BRAF* and *MYC* was dependent on ATF1 expression, but independent of BRCA1 expression (Fig. 5A). These data suggest that BRCA1 functions as a coactivator of ATF1 for a subset of genes regulated by ATF1, and BRCA1-C61G, but not C64G, retains the coactivator activity. Furthermore, we evaluated the coactivator activity of the BRCA1 variants M18T, H41R, and C44F, which show severe HRD and high sensitivity to olaparib (Fig. 1B and C). Similar to C64G, these variants did not rescue the mRNA level of *NRAS* and *BIRC2* as efficiently as wild-type BRCA1 in BRCA1-knockdown cells (Fig. 5B). The transcription of *NRAS* and *BIRC2* that was dependent on both BRCA1 and ATF1 was also observed in cells treated with olaparib (Fig. 5C).

### ATF1 Expression Level Determines the Sensitivity to Olaparib in Cells with HRD Caused by Depletion of Non-BRCA1 Factors

**FIGURE 6:**
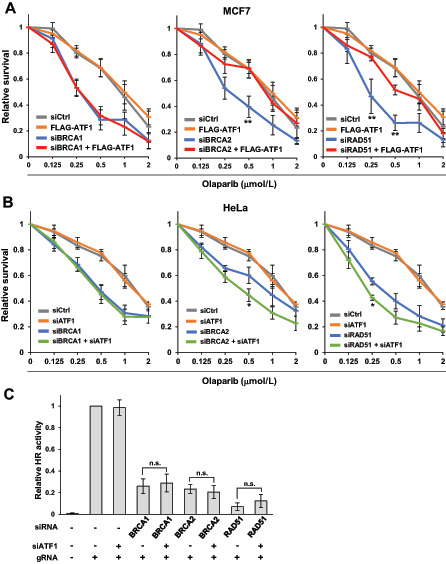
High ATF1 expression confers resistance to olaparib in non-BRCA1 HR factor–depleted cells. **A,** MCF7 cells were transfected as indicated and treated with olaparib for 5 days. Data are presented in three divided graphs for knockdown samples of BRCA1, BRCA2, or RAD51 for clarity, and they share the same data of siCtrl and siATF1 samples. Data represent the mean ± SEM of four independent experiments. **, *P* < 0.01 between knockdown of BRCA2 or RAD51 and knockdown of BRCA2 or RAD51 with FLAG-ATF1 overexpression. **B,** HeLa cells were transfected as indicated and treated with olaparib for 5 days. Data represent presented as in **A**. Data represent the mean ± SEM of four independent experiments. *, *P* < 0.05 between single knockdown of BRCA2 or RAD51 and double knockdown of BRCA2 or RAD51 and ATF1. **C,** HeLa cells were transfected as indicated, and HR activity was measured by ASHRA. Data represent the mean ± SEM of three independent experiments. n.s.: not significant.

BRCA1-C61G, which is HR deficient, but functions as a coactivator of ATF1-regulated transcription, conferred resistance to olaparib in cells with high ATF1 expression. Therefore, we speculated that a BRCA1/ATF1-mediated mechanism confers resistance to olaparib in HR-deficient cells because of the alteration of other HR factors. To confirm this hypothesis, we analyzed the effects of ATF1 overexpression on the sensitivity to olaparib in BRCA2- or RAD51-knockdown MCF7 (ATF1-low) cells (Supplementary Fig. S4A). ATF1 overexpression significantly conferred resistance to olaparib in BRCA2- or RAD51-knockdown MCF7 cells, but not in BRCA1-knockdown cells (Fig. 6A).

To confirm the ATF1-mediated resistance in cells with depletion of non-BRCA1 HR factors, we examined the effects of ATF1 knockdown on the sensitivity to olaparib in BRCA2- or RAD51-knockdown HeLa (ATF1-high) cells (Supplementary Fig. S4B). The sensitivity to olaparib was significantly increased by ATF1 knockdown in BRCA2- or RAD51-knockdown HeLa cells, but not in BRCA1-knockdown cells (Fig. 6B). Similar results were obtained in another ATF1-high cell line, BT-549 (Supplementary Fig. S4C). Although the expression levels of ATF1 had a mild effect on of BRCA1 and BRCA2 expression (Supplementary Fig. S4A and S4B), they did not markedly affect HR activity (Fig. 6C and data not shown). ATF1-regulated transcription of *NRAS* and *BIRC2* was observed in BRCA2- or RAD51-knockdown cells (Supplementary Fig. S4D).

### BRCA1/ATF1-Mediated Transactivation Confers Resistance to Cisplatin

**FIGURE 7:**
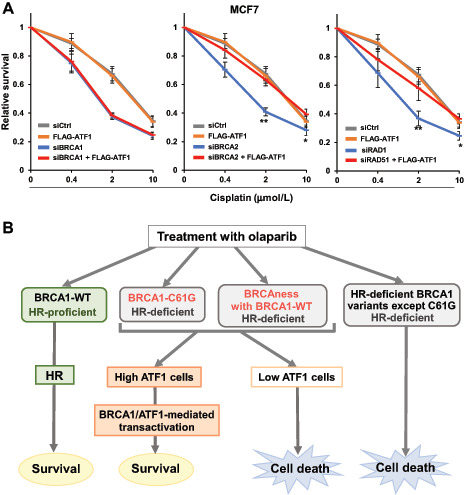
High ATF1 expression confers resistance to PARP inhibitors and platinum agents in non-BRCA1 HR factor–depleted cells. **A,** MCF7 cells were transfected as indicated and treated with cisplatin for 4 days. Data are presented as in Fig. 6A. Data represent the mean ± SEM of four independent experiments. **, *P* < 0.01; *, *P* < 0.05 between knockdown of BRCA2 or RAD51 and knockdown of BRCA2 or RAD51 with FLAG-ATF1 overexpression. **B,** Schematic of ATF1-dependent survival and cell death in HR-deficient cells. When treated with olaparib, HR-proficient cells can repair DNA damage induced by olaparib and cells survive. Cells with the C61G variant cannot efficiently repair DNA damage by HR; however, in cells with high ATF1 expression, the C61G variant activates ATF1-mediated transcription and promotes cell proliferation and survival. HR-deficient cells (BRCAness), which show altered non-BRCA1 HR factors, such as BRCA2 or RAD51, but possess wild-type BRCA1, activate ATF1-mediated transcription to support cell survival under conditions of high ATF1 expression. Cells with the HR-deficient BRCA1 variants except C61G, such as the C64G variant, cannot repair DNA damage and activate transcription with ATF1, resulting in cell death.

HRD also sensitizes cells to cisplatin. To investigate whether BRCA1/ATF1-mediated transactivation contributes the resistance to cisplatin, we analyzed the effect of ATF1 overexpression on the sensitivity to cisplatin in BRCA1-, BRCA2-, or RAD51-knockdown MCF7 cells. As shown in Fig. 7A, ATF1 overexpression significantly conferred resistance to cisplatin in BRCA2- or RAD51-knockdown cells, but not in BRCA1-knockdown cells.

## Discussion

In this study, we showed that HR activity measured by ASHRA was significantly correlated with sensitivity to olaparib in cells expressing BRCA1 missense variants (Fig. 2). The correlation was almost linear, and the correlation coefficient was as high as 0.88 in HeLa cells and 0.87 in MCF7 cells.

ASHRA detected intermediate HR activity, which was not detected in our previous analyses using by the DR-GFP assay (refs. 20, 21; Table 1). Among 30 variants analyzed, 18 (shown by yellow or pale pink bars in Fig. 1B) were categorized as HR-proficient by the DR-GFP assay. When measured by ASHRA, 13 of the 18 variants showed >80% HR activity of wild-type BRCA1. The remaining five variants, I21V, I31M, R71G, N132K, and S153R (shown by pale pink bars in Fig. 1B), possessed only 80% or lower HR activity. The D67Y variant (shown by pale pink bars) is categorized as benign in ClinVar (https://www.ncbi.nlm.nih.gov/clinvar/); however, the HR activity of BRCA1-D67Y was >80%, but significantly lower than that of wild-type BRCA1. This is consistent with the report that the D67Y variant decreases the activity of an E3 ubiquitin ligase and might affect the sensitivity to cisplatin *in vitro* (38).

**TABLE 1:**
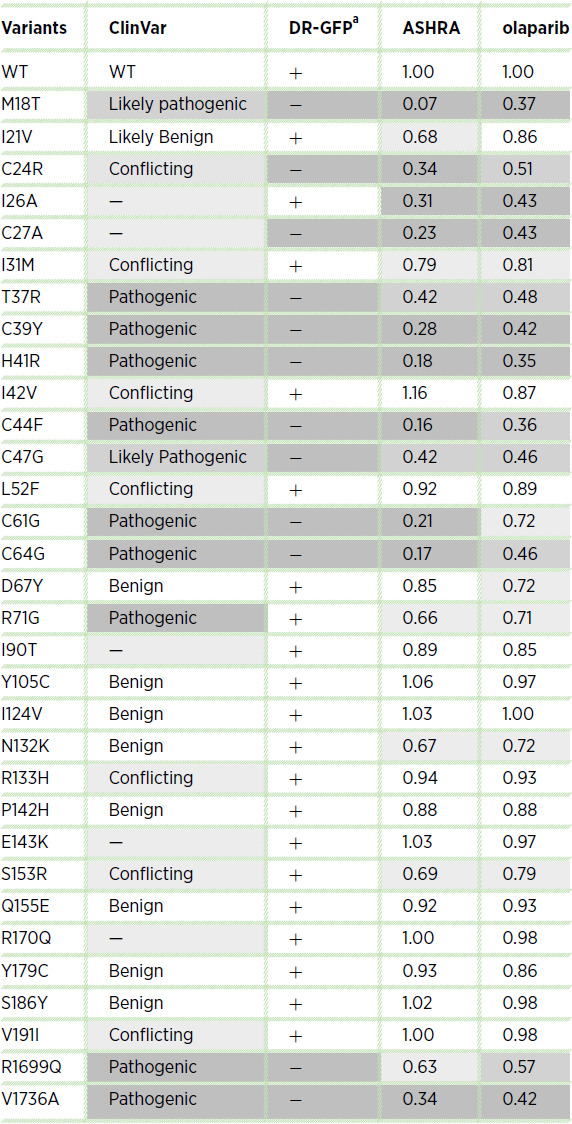
Summary of the missense variants of BRCA1 analyzed in this study

NOTE: Pathogenicity curated in ClinVar and previously reported data obtained with the DR-GFP assay (20–23) are also listed. ASHRA and olaparib columns show relative HR activity and survival in the presence of 0.5 mmol/L olaparib in HeLa cells expressing each variant. In the DR-GFP columns, white and dark gray cells indicate proficient and deficient cells, respectively. In the ASHRA and olaparib columns, white, pale gray, gray, and dark gray cells indicate first, second, third, and fourth quartiles, respectively.

^a^From references 20, 21, 22 and 23.

Among 10 variants categorized as HR deficient by the DR-GFP assay (shown by blue and pale blue bars), four (C24R, I26A, T37R, and C47G; shown by pale blue bars) showed low, but significantly higher HR activity than BRCA1 knockdown and other severely HR-deficient variants such as M18T, H41R, C61G, and C64G (shown by blue bars).

The R1699Q variant is associated with moderately increased cancer risk (28, 29). The V1736A variant is a hypomorphic variant that increases the risk of ovarian cancer through biallelic pathogenic variation (30). However, these variants are categorized as HR deficient by the DR-GFP assay (22, 23) despite these moderate phenotypes. In contrast, ASHRA clearly detected the intermediate HR activity of BRCA1-R1699Q and -V1736A (Fig. 2E), which was associated with intermediate sensitivity to olaparib (Fig. 2F and G) and cancer risk (27–30). These results suggest that HR activity determined by ASHRA could predict the cancer risk in addition to the sensitivity to PARP inhibitors. Although it is unclear how ASHRA can quantitatively evaluate HR activity, targeting endogenous gene loci and detecting HR products at the DNA level might contribute to the quantitative measurement. Five variants (I21V, I31M, R71G, N132K, and S153R) showed intermediate activity that was similar to that of the R1699Q variant (Figs. 1B and 2F). R71G is classified as a pathogenic variant, because it affects the splicing of *BRCA1* mRNA (39, 40). The I21V, I31M, N132K, and S153R variants are not classified as pathogenic (Table 1). Although BRCA1 has several functions as a tumor suppressor in addition to HR activity (41), these four variants might confer moderate cancer risk similar to the R1699Q variant.

In this study, we determined the effect of 12 BRCA1 variants with conflicting or no information in ClinVar on HR activity by ASHRA (shown by pale gray in Table 1). Seven variants (I42V, L52F, I90T, R133H, E143K. R170Q, and V191I) were HR proficient, and two variants (I31M and S153R) were partially HR proficient. Three variants (C24R, I26A, and C27A) were HR deficient or partially HR-deficient and sensitive to olaparib. These data may help clinical decision making in individuals carrying these variants.

The present data, however, may lead to problems associated with patient stratification for treatment with PARP inhibitors. The BRCA1 variants T37R and C47G, which are classified as pathogenic or likely pathogenic in ClinVar or nonfunctional by the saturation gene editing using HAP1 cells (42), had intermediate HR activity according to ASHRA (Table 1; Fig. 1B). This intermediate HR activity conferred partial resistance to olaparib in cells expressing these variants (Fig. 1C), which may contribute to the resistance to a PARP inhibitor observed clinically. Conversely, the variants I21V, I31M, N132K, and S153R, which are classified as benign, likely benign, or conflicting, showed partial HRD and moderate sensitivity to olaparib. Therefore, the treatment of patients carrying variants with partial HRD could be an important clinical issue.

HR activity determined by ASHRA was highly correlated with the sensitivity to olaparib in both HeLa and MCF7 cells, whereas HeLa cells expressing BRCA1-C61G were disproportionately resistant to olaparib despite showing severe HRD (Fig. 2A and B). Homozygous C61G mice are embryonic lethal and develop spontaneous tumors similar to BRCA1 null mice, whereas mouse tumor cells carrying the C61G variant show a poor response to olaparib and cisplatin and rapidly develop resistance (43). In addition, some cisplatin-resistant tumors with the C61G variant show increased expression of BRCA1-C61G (43), and BRCA1-C61G has residual activity in the DNA damage response (44). These data suggest that BRCA1 C61G is a hypomorphic variant associated with a high cancer risk and retains some activity to protect cells from PARP inhibitors or cisplatin.

ATF1 expression was higher in HeLa and BT-549 cells than in MCF7 and MCF10A cells (Fig. 3B). Although the results were obtained only four cell lines, overexpression of ATF1 decreased the sensitivity to olaparib in MCF7 and MCF10A (i.e., ATF1-low) cells expressing BRCA1-C61G (Fig. 3C–E), and knockdown of ATF1 significantly increased the sensitivity to olaparib in HeLa and BT-549 (i.e., ATF1-high) cells expressing BRCA1-C61G (Fig. 4B; Supplementary Fig. S4B). BRCA1-C61G, but not BRCA1-C64G, conferred resistance to olaparib under conditions of high ATF1 expression (Fig. 7B). ATF1 expression levels did not significantly affect HR activity (Figs. 4C, 6C; Supplementary Fig. S3C). These findings suggest that the resistance to olaparib of BRCA1-C61G–expressing cells with high ATF1 expression was not related to the recovery of HR activity.

ATF1 positively regulates cell proliferation and survival by regulating the transcription of genes involved in the MAPK, Wnt, and NFκB pathways (26, 36). Thus, activation of ATF1 may support cell survival in the presence of olaparib. We found that the BRCA1-C61G variant interacts with ATF1 and activates ATF1-mediated transcription; however, this was not observed in the BRCA1-C64G variant, which has similar functions to those of BRCA1-C61G, except for its role as a coactivator of ATF1 (Fig. 5; Supplementary Table S1). Furthermore, other variants with severe HRD, such as M18T, H41R, and C44F, did not activate ATF1-mediated transcription, similar to BRCA1-C64G variant. These data suggest that the BRCA1-C61G variant could exceptionally function as a coactivator for ATF1-regulated transcription among the analyzed HR-deficient variants.

Two types of ATF1-target genes were identified: BRCA1-dependent (*NRAS* and *BIRC2*) and BRCA1-independent (*BRAF* and *MYC*) genes (Fig. 5A). It remains unclear how BRCA1 differentially regulates a certain subset of transcription of ATF1-taget genes. Thus, BRCA1-C61G, but not other HR-deficient variants, might activate the transcription of a certain subset of ATF1 target genes in the presence of sufficient ATF1, resulting in resistance to olaparib (Fig. 7B). It should be elucidated which genes co-operatively regulated by BRCA1 and ATF1 are responsible for the resistance to PARP inhibitors. The identification of the responsible genes might lead to the development of biomarkers of response to PARP inhibitors and therapeutics capable of reverting the resistance to PARP inhibitors.

We presumed that high ATF1 expression confers resistance to PARP inhibitors in cancer cells with HRD because of the alteration of non-BRCA1 HR factors, such as BRCA2, in which sufficient ATF1 and intact BRCA1 could stimulate ATF1-mediated transactivation. As shown in Fig. 6, high expression of ATF1 conferred resistance to olaparib in BRCA2-knockdown or RAD51-knockdown cells. Importantly, ATF1 knockdown or overexpression did not affect the sensitivity to olaparib in BRCA1-knockdown cells. High expression of ATF1 also conferred resistance to cisplatin in BRCA2-knockdown or RAD51-knockdown cells (Fig. 7A) and in cells expressing BRCA1-C61G (data not shown), but not in BRCA1-knockdown cells (Fig. 7A), suggesting that a BRCA1/ATF1-mediated mechanism may also confer resistance to platinum agents in HR-deficient cells. In ATF1-low MCF7 cells, knockdown of BRCA1, BRCA2, or RAD51 resulted in similar sensitivity to olaparib and cisplatin, whereas BRCA2-knockdown or RAD51-knockdown cells became resistant to these agents by overexpressing ATF1 compared with BRCA1-knockdown cells (Supplementary Fig. S5A and S5B). In ATF1-high HeLa cells, BRCA2-knockdown cells seemed to be more resistant than BRCA1-knockdown cells, whereas double knockdown with ATF1 increased sensitivity to olaparib in BRCA2-knockdown cells, resulting in similar sensitivity to that of BRCA1-knockdown cells (Supplementary Fig. S5C). The effect of RAD51 knockdown was strong in HeLa cells, and sensitivity to olaparib was similar between RAD51-knockdown and BRCA1-knockdown cells; double knockdown of RAD51 and ATF1 further increased the sensitivity to olaparib (Supplementary Fig. S5C). Thus, the expression level of ATF1 may be an important biomarker to predict the efficacy of therapeutics in patients with BRCAness tumors harboring alterations of non-BRCA1 HR factors, as well as in patients carrying the BRCA1-C61G variant.

Because this study does not contain clinical data, the clinical significance of the BRCA1/ATF1-dependent resistance to PARP inhibitors and platinum agents remains to be elucidated. ATF1 overexpression was reported in many cancer types including adrenal, esophageal, ovarian, and breast cancers, as well as in soft-tissue sarcoma (COSMIC, https://cancer.sanger.ac.uk/cosmic/gene/analysis?ln=ATF1). To our knowledge, there is no study that investigated the significance of ATF1 expression in the effect of cancer treatment with PARP inhibitors or platinum agents.

This study suggests that BRCA1/ATF1-dependent resistance to PARP inhibitors and platinum agents should be considered in patients harboring a certain BRCA1 mutation and the deficiency of non-BRCA1 HR factors. BRCA1-C61G is a founder mutation in the Polish population and is included in standard panel tests for the diagnosis of hereditary cancer and for the treatment of breast and ovarian cancers in Polish patients (45, 46). In patients with ovarian cancer, alterations in non-BRCA1 factors are involved in more than half of all cases with HRD (9). Therefore, resistance to PARP inhibitors and platinum agents induced by high ATF1 expression may be of particular importance for these populations and these should be further investigated in future studies.

Furthermore, it is important question whether BRCA1/ATF1-mediated transactivation also confers resistance to chemotherapeutics other than PARP inhibitors and platinum agents. Cheng and colleagues reported gene amplification of the chromosome region containing *ATF1* could be a predictive biomarker for high sensitivity of sarcoma cell lines to several chemotherapeutics including alkylating agents and nucleoside analogues, but not bleomycin (47).

The fusion gene of *ATF1* with *EWSR1* or *FUS* is frequently reported in sarcomas including angiomatoid fibrous histiocytoma and clear cell sarcoma, which are relatively chemoresistant (48, 49). EWSR1 and FUS are multifunctional proteins that belong to the FET family of RNA-binding proteins. The *EWSR1/ATF1* fusion gene product, which consists of the N-terminal transactivation domain of EWSR1 and the C-terminal DNA binding domain of ATF1, has enhanced transcriptional activity and functions as a key driver oncogene (50, 51). Thus, these fusion gene products may be involved in the resistance to chemotherapy in sarcomas. Future studies should investigate the role of ATF1 translocation in the resistance to PARP inhibitors or DNA-damaging agents in these malignancies.

In conclusion, we showed that ASHRA could quantitatively measure HR activity, which is correlated to the sensitivity to PARP inhibitors and cancer risk. The use of ASHRA led to the identification of high ATF1 expression as a potential mechanism of resistance to PARP inhibitors and platinum agents. This mechanism is unique because it depends on the interaction between BRCA1 and ATF1 irrespective of the HR activity of BRCA1, and it could become a new target of therapeutic strategies. Quantitative analysis by ASHRA may contribute to the investigation of DNA damage repair pathways and improve patient stratification for treatment with PARP inhibitors and platinum agents.

## Supplementary Material

Table S1 Summary of the effects of BRCA1 variants on BRCA1 functions

Figure S1 Analysis of 30 BRCA1 missense variants in HeLa cells

Figure S2 Analysis of 30 BRCA1 missense variants in MCF7 cells

Figure S3 Contribution of ATF1 to olaparib resistance in BT-549 cells

Figure S4 Contribution of ATF1 to olaparib resistance in BRCA2- or RAD51-knockdown cells

Figure S5 Effects of ATF1 expression level on sensitivity in BRCA1-, BRCA2-, or RAD51-knockdown cells
