# Supplementary material for: BRCA1/ATF1-Mediated Transactivation is Involved in Resistance to PARP Inhibitors and Cisplatin
Source: Cancer Res Commun. 2021 Nov 12;1(2):90–105. doi: 10.1158/2767-9764.CRC-21-0064 (PMC9973406; doi:10.1158/2767-9764.CRC-21-0064)
Supplement: Table S1 — Summary of the effects of BRCA1 variants on BRCA1 functions [file crc-21-0064-s01.pdf]

# Supplementary Table 1

| BRCA1 | WT | C61G | C64G |
|---|---|---|---|
| **Binding to BARD1** | + | - | - |
| **E3 ligase activity** | + | - | - |
| **HR activity** | + | - | - |
| **Resistance to Olaparib in HeLa cells** | + | + | - |
| **Activation of ATF1** | + | + | - |

**Supplementary Table S1. Summary of the effects of BRCA1 variants on BRCA1 functions**
