# Supplementary material for: BRCA1/ATF1-Mediated Transactivation is Involved in Resistance to PARP Inhibitors and Cisplatin
Source: Cancer Res Commun. 2021 Nov 12;1(2):90–105. doi: 10.1158/2767-9764.CRC-21-0064 (PMC9973406; doi:10.1158/2767-9764.CRC-21-0064)
Supplement: Figure S1 — Analysis of 30 BRCA1 missense variants in HeLa cells [file crc-21-0064-s02.pdf]

Supplementary Figure S1

A

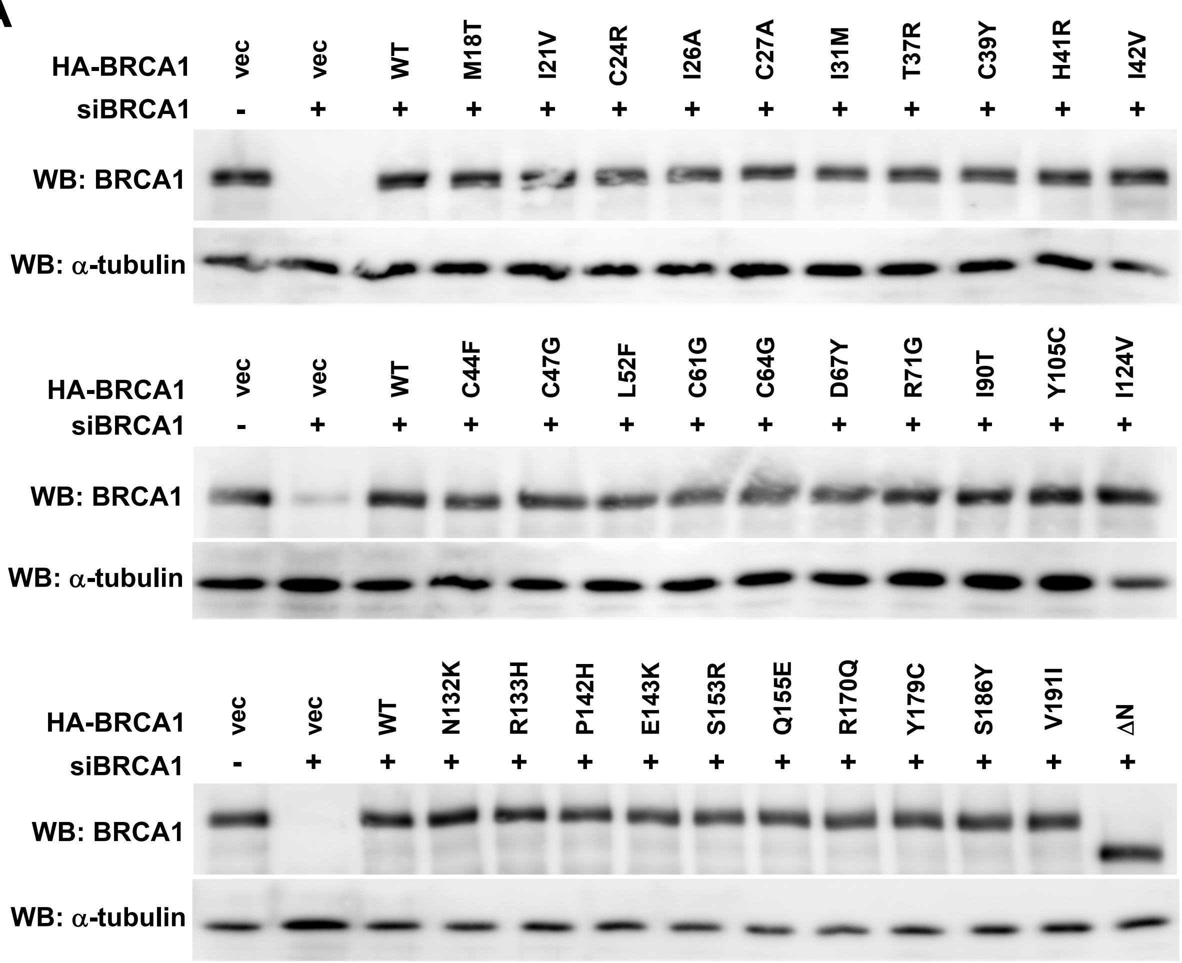

B

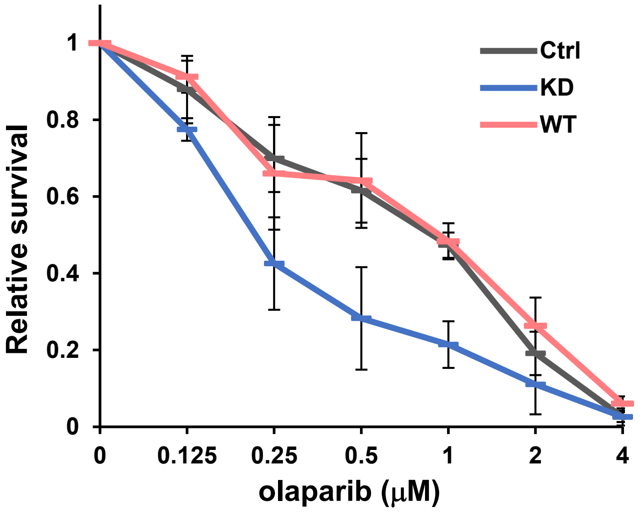

Supplementary Figure S1. Analysis of 30 BRCA1 missense variants in HeLa cells

A, Whole cell lysates of the samples in Fig. 1C were analyzed by western blotting. **B,** HeLa cells were treated with the indicated concentrations of olaparib for 5 days. Data represent the mean ± SEM of three independent experiments. Ctrl: cells transfected with the non-target siRNA and the empty vector; KD: cells transfected with the target siRNA and the empty vector; WT: cells transfected with the target siRNA and the wild-type BRCA1 expression vector.
