# Supplementary material for: BRCA1/ATF1-Mediated Transactivation is Involved in Resistance to PARP Inhibitors and Cisplatin
Source: Cancer Res Commun. 2021 Nov 12;1(2):90–105. doi: 10.1158/2767-9764.CRC-21-0064 (PMC9973406; doi:10.1158/2767-9764.CRC-21-0064)
Supplement: Figure S3 — Contribution of ATF1 to olaparib resistance in BT-549 cells [file crc-21-0064-s04.pdf]

Supplementary Figure S3

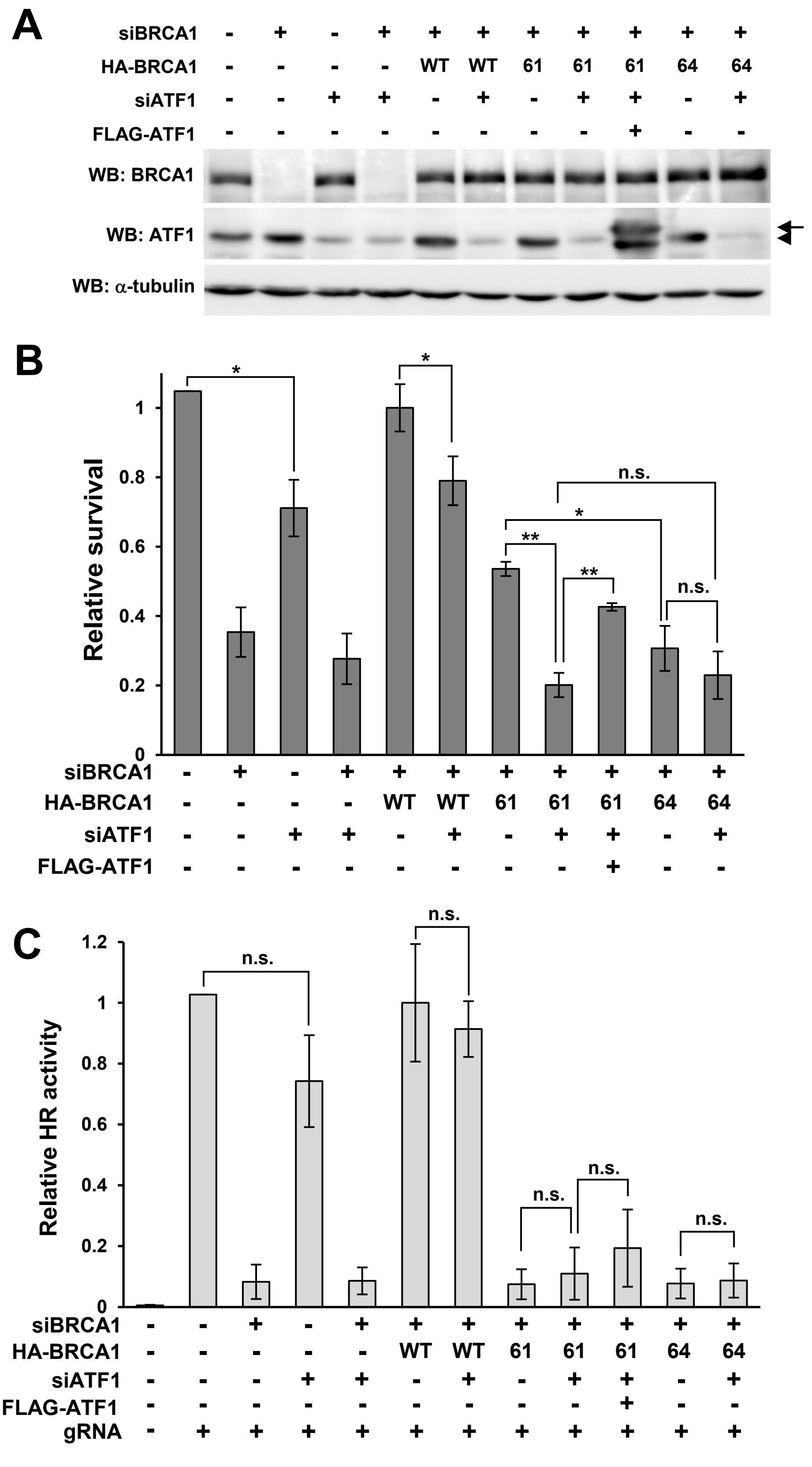

Supplementary Figure S3. Contribution of ATF1 to olaparib resistance in BT-549 cells
A, BT-549 cells were transfected as indicated, and whole cell lysates were analyzed by western blotting. Arrowhead: endogenous ATF1; Arrow: FLAG-ATF1. **B,** BT-549 cells were transfected as indicated and treated with 4.0 mM olaparib for 5 days. WT, 61, and 64 indicate BRCA1-wild type, -C61G, and -C64G, respectively. Data represent the mean ± SEM of three independent experiments. *: $P < 0.05$ **: $P < 0.01$, n.s.: not significant. **C,** BT-549 cells were transfected as indicated, and HR activity was measured by ASHRA. Data represent the mean ± SEM of three independent experiments. n.s.: not significant.
