# Supplementary material for: BRCA1/ATF1-Mediated Transactivation is Involved in Resistance to PARP Inhibitors and Cisplatin
Source: Cancer Res Commun. 2021 Nov 12;1(2):90–105. doi: 10.1158/2767-9764.CRC-21-0064 (PMC9973406; doi:10.1158/2767-9764.CRC-21-0064)
Supplement: Figure S4 — Contribution of ATF1 to olaparib resistance in BRCA2- or RAD51-knockdown cells [file crc-21-0064-s05.pdf]

Supplementary Figure S4

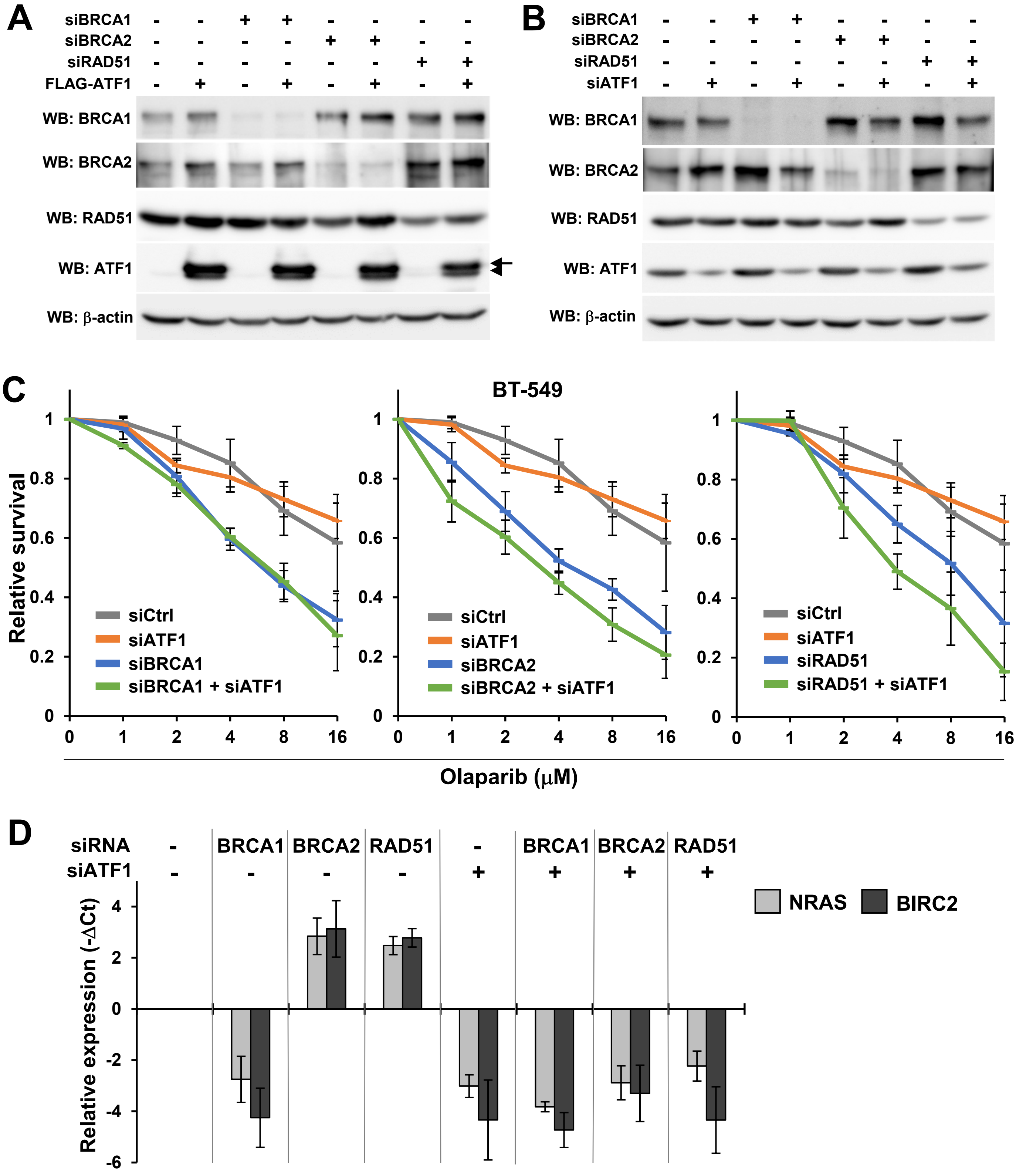

Supplementary Figure S4. Contribution of ATF1 to olaparib resistance in BRCA2- or RAD51-knockdown cells

A, B, MCF7 or HeLa cells were transfected as indicated and harvested after 72 h of incubation. Whole cell lysates were analyzed by western blotting. **C,** BT-549 cells were transfected as indicated and treated with olaparib for 5 days. Data are presented in three divided graphs for knockdown samples of BRCA1, BRCA2, or RAD51 for clarity, and they share the same data of siCtrl and siATF1 samples. Data are expressed as the mean ± SEM of four independent experiments. *: $P < 0.05$. **D,** HeLa cells were transfected as indicated and mRNA levels of *NRAS* and *BIRC2* were quantified by RT-qPCR 72 h after transfection. Data represent the mean ± SEM of three independent experiments.
