# Supplementary material for: BRCA1/ATF1-Mediated Transactivation is Involved in Resistance to PARP Inhibitors and Cisplatin
Source: Cancer Res Commun. 2021 Nov 12;1(2):90–105. doi: 10.1158/2767-9764.CRC-21-0064 (PMC9973406; doi:10.1158/2767-9764.CRC-21-0064)
Supplement: Figure S5 — Effects of ATF1 expression level on sensitivity in BRCA1-, BRCA2-, or RAD51-knockdown cells [file crc-21-0064-s06.pdf]

# Supplementary Figure S5

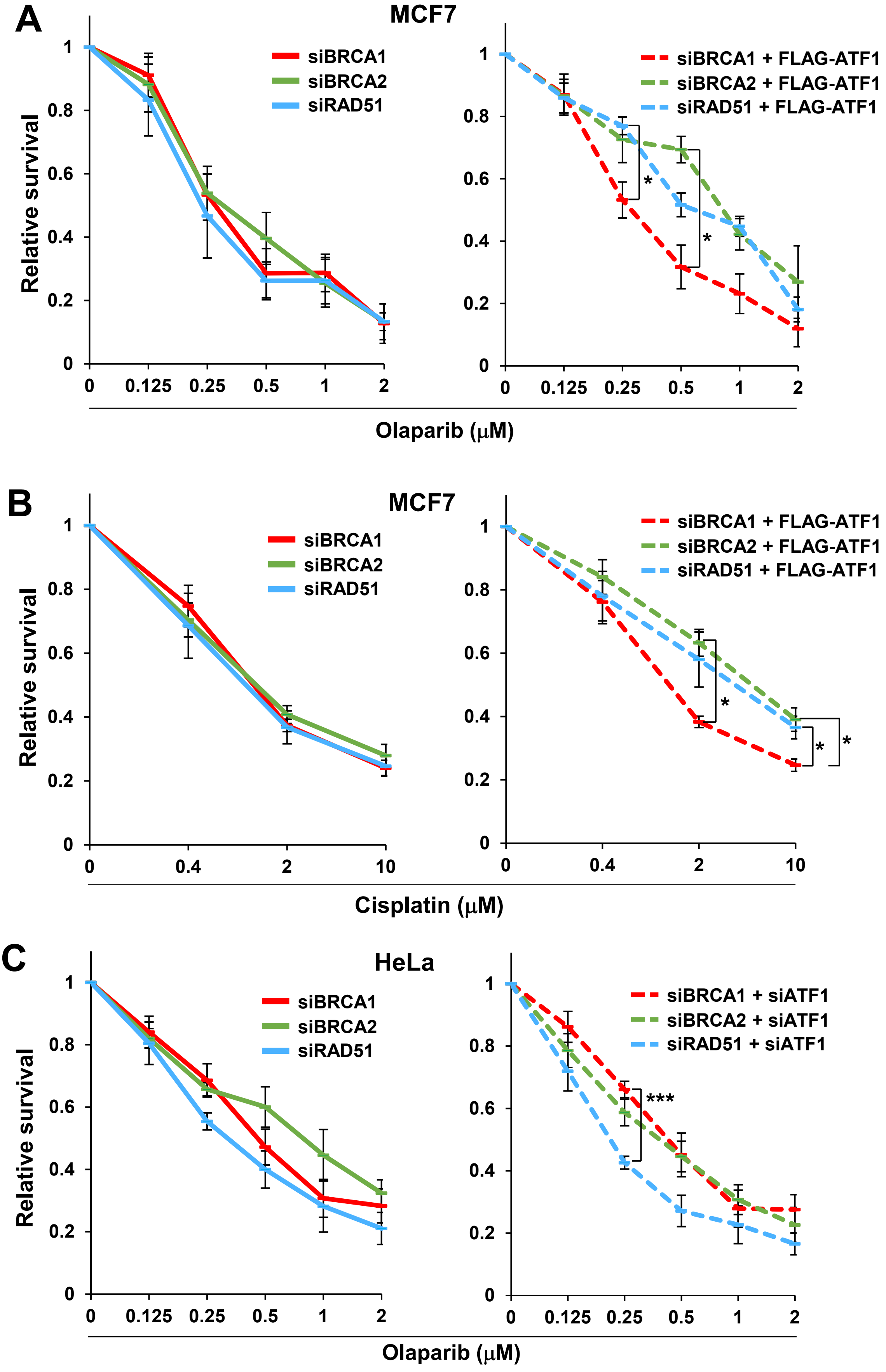


**Supplementary Figure S5. Effects of ATF1 expression level on sensitivity in BRCA1-, BRCA2-, or RAD51-knockdown cells**

**A, B, C,** Data of Figs. 6A, 7A, and 6B were replotted. Data represent the mean ± SEM of four independent experiments. The solid lines represent single knockdown of BRCA1, BRCA2, or RAD51, and the broken lines represent concomitant overexpression (A and B) or knockdown (C) of ATF1.
